# The Prevalence of Tobacco and E-Cigarette Use in Poland: A 2019 Nationwide Cross-Sectional Survey

**DOI:** 10.3390/ijerph16234820

**Published:** 2019-11-30

**Authors:** Jarosław Pinkas, Dorota Kaleta, Wojciech Stefan Zgliczyński, Aleksandra Lusawa, Iwona Wrześniewska-Wal, Waldemar Wierzba, Mariusz Gujski, Mateusz Jankowski

**Affiliations:** 1 School of Public Health, Centre of Postgraduate Medical Education, 01-826 Warsaw, Poland; jaroslaw.pinkas@cmkp.edu.pl (J.P.); iwrzesniewska@cmkp.edu.pl (I.W.-W.); mateusz.jankowski@cmkp.edu.pl (M.J.); 2 Chief Sanitary Inspectorate, 03-729 Warsaw, Poland; a.lusawa@gis.gov.pl; 3 Department of Hygiene and Epidemiology, Medical University of Lodz, 90-752 Łódź, Poland; dorota.kaleta@umed.lodz.pl; 4 UHE Satellite Campus in Warsaw, University of Humanities and Economics in Łódź, 01-513 Warsaw; Poland; wwierzba@post.pl; 5 Department of the Prevention of Environmental Hazards and Allergology, Medical University of Warsaw, 02-091 Warsaw, Poland; mariusz.gujski@wum.edu.pl

**Keywords:** tobacco, smoking, e-cigarettes, heated tobacco, epidemiology, Poland

## Abstract

Monitoring of tobacco use is one of the key tobacco control activities. This study aimed to assess the current prevalence and patterns of tobacco and e-cigarette in Poland as well as to investigate socioeconomic factors associated with cigarette smoking and e-cigarette use. This cross-sectional study was carried out in 2019, on a representative nationwide sample of 1011 individuals aged 15+ in Poland. Daily tobacco smoking was declared by 21.0% of participants; 1.3% of participants were occasional tobacco smokers, and 10.7% were former tobacco smokers. Heated tobacco was used by 0.4% of participants. Ever e-cigarette use was declared by 4.0% of participants and 1.4% were current e-cigarette users. A higher proportion of daily smokers was observed among men than women (24.4% vs. 18.0%; *p* < 0.0001). The age group 30 to 49 years, of a lower educational level and living in a medium-sized city (between 20,000 and 500,000 residents), was significantly associated with current daily smoking. This is the most up-to-date study on the prevalence of smoking in Poland. Further tobacco control activities are needed to reduce tobacco use in Poland.

## 1. Introduction

Tobacco use, as well as exposure to secondhand tobacco smoke, are one of the most preventable causes of death and disability globally [1,2,3]. Tobacco smoking is a major risk factor for many noncommunicable diseases (NCDs) such as cancer, respiratory diseases, coronary artery disease and stroke [4,5]. According to World Health Organization (WHO) estimates, tobacco kills more than 8 million people globally each year, wherein around 1.2 million are the result of passive smoking (exposure to secondhand smoke) [6]. In the European Union (EU), almost 0.7 million deaths per year are related to tobacco use [7]. In Poland, tobacco use is the leading cause of preventable death and is responsible for approximately 70,000 deaths annually [2]. The global economic cost of smoking-attributable diseases is equivalent to 1.8% of the world’s annual gross domestic product [8]. The European Commission estimated that smoking costs the EU countries at least €100 billion per year [7].

According to the WHO, 1.1 billion people globally smoke [6]. An analysis of global trends in tobacco use showed that between 2000 and 2010, the prevalence of tobacco smoking in men fell in 125 of 173 analysed countries, and in women, fell in 155 of 178 analysed countries [9]. The prevalence of smoking has also decreased in Europe [10]. This is mainly related to increased awareness of the health effects of tobacco use as well as more restrictive national tobacco control regulations [9,10].

According to the Special Eurobarometer 458 survey, in 2017, over a quarter (26%) of EU citizens were smokers [7]. The proportion of smokers in the EU has been stable since 2014 [7]. However, there were significant differences in the prevalence of tobacco use across the EU, with the highest proportion rates of smoking in Southern Europe [5,10,11]. The prevalence of smoking in Central and Eastern Europe was also higher, compared to the EU average [5,7]. In 2017, Poland was the sixth country out of 28 EU countries in terms of the frequency of smoking [7]. 

In recent years, alternative forms of nicotine delivery such as e-cigarettes and heated tobacco are gaining popularity [12,13]. Between 2017 and 2014, the proportion of Europeans who had ever tried an e-cigarette increased by 21% [13]. It is estimated that 2% of EU citizens are regular e-cigarette users [7,13]. 

The WHO Framework Convention on Tobacco Control (FCTC) requires the Member States to consistently collect national data on the magnitude, patterns, and determinants of tobacco use [14]. It is believed that monitoring is the foundation of successful tobacco control [14,15]. The last nationwide cross-sectional survey on tobacco use in Poland was carried out in 2017 by Chief Sanitary Inspectorate [16]. Due to the lack of current epidemiological data on the frequency of smoking and the use of e-cigarettes, this study aimed to assess (1) the current prevalence and patterns of tobacco and e-cigarette use as well as (2) to investigate socioeconomic factors associated with cigarette smoking and e-cigarette use.

## 2. Materials and Methods

### 2.1. Study Design and Population 

This cross-sectional study was carried out between 20 and 25 September 2019 on a representative nationwide sample of 1011 individuals aged 15+ in Poland. The computer-assisted personal interviewing (CAPI) technique was used [17]. A random quota sample was selected from the National Official Register of the Territorial Division of the Country (TERYT; address-based) sampling frame [18]. The stratification model includes gender, age, as well as the size of domicile and the territorial distribution within voivodships. The stratification was based on demographic data from the “Population Report. Status and structure in territorial division.” published annually by the Central Statistical Office of Poland [18]. A random quota sample used for this survey ensured a random selection of locations for the survey and guarantees that the sample structure corresponds with the structure of population. All the interviews were carried out by a specialized survey company —Kantar—on behalf of the Chief Sanitary Inspectorate (GIS), which provides the context of this research.

Participation in the study was voluntary and anonymous. Participants had the right to refuse to participate without giving a reason. All the procedures performed in studies involving human participants were in accordance with the ethical standards of the institutional and/or national research committee and with the 1964 Helsinki Declaration and its later amendments or comparable ethical standards. According to the current guidelines of the Ethical Review Board at the Centre of Postgraduate Medical Education, Warsaw, Poland, an anonymous questionnaire-based cross-sectional study does not require separate consent.

Participants in the survey were divided according to their self-declared smoking status into the following groups: daily smokers, occasional smokers, former smokers and nonsmokers (without smoking history). 

### 2.2. Study Questionnaire 

The research tool was an original questionnaire developed for the purpose of this study. In preparation of the questionnaire, we analysed the previously published nation-wide cross-sectional surveys about attitudes towards tobacco consumption [7,19,20,21], with special emphasis on the Global Adult Tobacco Survey (GATS) [20,21]. The questionnaire included 42 questions related to the frequency and patterns of cigarettes, e-cigarettes, and heated tobacco use. Questions also addressed personal characteristics, including gender (male or female), age (years), marital status, place of residence, education level, occupational status as well as financial situation. In the case of the age criterion, the following was applied: 15–19 years, 20–29 years, 30–39 years, 40–49 years, 50–59 years and 60+ years. Marital status was classified as single, married, divorced, or widowed. Place of residence was classified as follows: rural, city up to 20,000 residents, city between 20,000–100,000 residents, city between 100,000–500,000 residents, city above 500,000 residents. Educational level was classified as primary education, vocational education, secondary education or higher education. The occupational activity was classified as active (currently employed) or passive (currently unemployed) occupational status. The financial situation was assessed according to the following measures: good (sufficient to cover all living needs and able to save a certain amount), moderate (sufficient to cover all living needs), bad (not sufficient to cover even the basic needs).

Smoking status was defined according to the answers to the questions: “Have you ever smoked at least 100 cigarettes (or similar amount of other tobacco products e.g., pipes, cigars, cigarillos) in your lifetime?” and “Do you currently smoke?”. Current smokers were respondents who reported having smoked ≥100 cigarettes (or similar amount of other tobacco products) during their lifetime. Moreover, based on the answer to the question: “During the past six months, have you smoked tobacco daily?”, this group was divided into “daily” smokers or “occasional” smokers. Former smokers were respondents who reported having smoked ≥100 cigarettes (or other tobacco products) during their lifetime but were not smoking at the time of the study. Non-smokers were respondents who reported having smoked fewer than 100 cigarettes (or other tobacco products) during their lifetime and who do not smoke now. The mean number of cigarettes or other tobacco products smoked per day was calculated among daily smokers. Ever or current e-cigarette use was defined according to the answers to the questions: “Have you ever used/tried an e-cigarette?” and “Do you currently use an e-cigarette?”.

### 2.3. Statistical Analysis 

The data were analysed with SPSS version 25 (IBM, Armonk, NY, USA). Normality of distributions of continuous variables was assessed by the Shapiro–Wilk test. Statistical significance of differences between continuous variables was analysed by the independent samples t-test or if the assumptions for this were not met, the Mann–Whitney U test was used. The distribution of categorical variables was shown by frequencies and proportions along with 95% confidence intervals. Statistical testing to compare categorical variables was completed using the independent samples chi-square test. 

Associations between personal characteristics (gender, age, marital status, place of residence, educational level, occupational status, and financial situation) with smoking status were conducted using the logistic regression analyses. Daily smoking, ever e-cigarette use and current e-cigarette use were considered separately as a dependent variable in the model. The socio-demographic characteristics (gender, age, marital status, place of residence, educational level, occupational status, and financial situation) were considered as independent variables. In univariate logistic regression analyses, all variables were considered separately. Multivariate logistic regression analyses included all of the variables significantly associated with the physicians’ behaviors toward cigarette or e-cigarette use by the patients in any of the univariate models (*p* < 0.05). The strength of association was measured by the odds ratio (OR) and 95% confidence intervals (CI). Statistical inference was based on the criterion *p* < 0.05.

## 3. Results

### 3.1. Characteristics of the Study Population 

The analysis is based on responses to survey forms received from 1011 people (52.1% females). Table 1 shows the characteristics of the sample classified by smoking status, separately for men and women. 

The prevalence of smoking was 19.2% among females and 25.8% among males (*p* = 0.01). In relation to females, the highest prevalence of smoking was observed among those aged 30–39 years and 50–59 years (Table 1). Divorced women smoked the most often, compared to married, single, or widowed (*p* = 0.001). The women who lived in cities between 20,000 and 500,000 citizens smoked the most (*p* < 0.01). For males, the highest prevalence of smoking was observed in the age group 40–49 years (Table 1). Among both men and women, those with vocational education smoked more often compared to participants with primary, secondary or higher education (*p* < 0.05). Details are presented in Table 1.

### 3.2. Smoking Prevalence and Patterns 

The prevalence of tobacco use is presented in Table 2. Among the participants, 21.0% were current daily tobacco smokers, 1.3% were current occasional tobacco smokers, and 67.0% were non-smokers. A higher proportion of daily smokers was observed among men than women (24.4% vs. 18.0%; *p* < 0.0001). Most of the daily smokers (76.5%) smoked regular cigarettes and 21.6% smoked hand-rolled cigarettes (Table 2). Slim or menthol cigarettes were smoked by 6.1% and 5.2% respectively. Women smoked slim (11.6% vs. 1.7%; *p* = 0.003) or menthol (8.4% vs. 2.5%; *p* = 0.04) cigarettes more often than men. Heated tobacco products were used by 1.9% of daily smokers (0.4% of all participants). Moreover, only 0.6% of all participants used smokeless tobacco. The mean starting age for daily smoking was 19.6 ± 4.6. Women started smoking at a later age than men (20.5 ± 4.4 years vs. 19.1 ± 4.5 years; *p* = 0.01). The participants who smoked daily smoke an average of 15 regular cigarettes a day, without significant differences (*p* > 0.05) between men and women (Table 2). Most of the participants had never tried an e-cigarette (96.0%). Ever e-cigarette use was declared by 4.0% and 1.4% were current e-cigarette users (Table 3). Among current e-cigarette users, 28.6% (*n* = 4) also smoked cigarettes on a daily basis (dual use). Men had tried e-cigarettes (5.6%) more often than women (3.0%; *p* = 0.04). The highest prevalence of ever and current e-cigarette use was observed in the city between 100,000–500,000 residents and the largest cities above 500,000 residents (Table 3). 

### 3.3. Associates of Smoking Status

The results of the univariate and multivariate regression analyses are presented in Table 3. Several characteristics, such as age between 30 to 49 years, lower educational level and living in a medium-sized city (between 20,000 and 500,000 residents), were significantly associated with current daily smoking among the Poles (Table 3). Participants aged 20–29 years (OR = 3.08; 95%CI: 1.09–8.68; *p* < 0.05) or 30–39 years (OR = 3.43; 95%CI: 1.29–9.16; *p* < 0.05) as well as those living in a city between 100,000 and 500,000 residents (OR = 6.75; 95%CI: 2.65–17.18; *p* < 0.001) or a city above 500,000 residents (OR = 3.56; 95%CI: 1.17–10.91; *p* < 0.05) had much higher odds of ever use of e-cigarette. 

## 4. Discussion

Tobacco use is a global public health problem [9,10]. Comprehensive tobacco control policies are key for reducing the prevalence of smoking both at local and international levels [14,15]. The implementation of the WHO Framework Convention on Tobacco Control (WHO FCTC) and MPOWER measures are a basic tool that helps countries reduce demand for tobacco [14,15]. “Monitoring tobacco use and prevention policies” is the first point of six MPOWER measures [15]. Monitoring tobacco use on a regular schedule allows the evaluation of tobacco control activities as well as the identification of specific risk groups [11,14,15]. A large number of epidemiological studies are assessing the frequency of smoking in individual European countries [7,10,11,13,21]. Nevertheless, several analyses have shown that monitoring of trends in tobacco use in European countries at national and EU levels is inconsistent, unstandardized, and in many cases, infrequent [11,22]. Tobacco consumption in the EU is regularly monitored with the Eurobarometer survey [7]. The Eurobarometer is a cross-sectional survey performed in a representative sample of the population of European Union (EU) member states [7]. There are several Special Eurobarometers on tobacco use in the EU member states published periodically [7,23,24]. Due to the use of a common methodology, these data provide a comparison between 28 member states [23]. Nevertheless, many countries, including Poland, conduct regular monitoring of tobacco use [11,16]. Since 2009, the Chief Sanitary Inspectorate has regularly published the results of a nation-wide survey about attitudes towards tobacco consumption towards smoking [16]. The results of the survey on Poles’ attitudes towards smoking in 2019 were the basis for the analyses presented in this study.

This is the most up-to-date epidemiological study on the prevalence of tobacco and e-cigarette use on a representative nationwide sample of Poles aged 15+ years. Moreover, this is the first study aimed at the prevalence of heated tobacco use in Poland. This study showed a decrease in the prevalence of smoking for both men and women. In 2017, 24% of Poles aged 15+ years smoked regularly [16]. In 2019, a 3% decrease in daily tobacco use was observed. Between 2017 [16] and 2019, the prevalence of daily smoking decreased from 29% to 24% among men and from 20% to 18% among women. The proportion of current smokers in Poland in this study is also lower, compared to previously reported in the Special Eurobarometer 458 (march 2017) [7]. According to the Special Eurobarometer 458, 30% of Poles were current smokers, wherein the prevalence of tobacco use was higher among men (34%) than women (26%) [7]. Currently, there is a lack of studies carried out in 2019, on a representative sample of EU citizens, therefore, international comparisons are not yet possible. Based on the multi-centre national population health examination survey (WOBASZ), the prevalence of tobacco smoking in Poland in the years from 2003 to 2014 decreased by 9% among men and by 4% among women [25]. According to the WOBASZ II study, the prevalence of smoking in 2014 was 29.9% among men and 20.5% among women [25]. The results of our study indicate a steady decrease in the frequency of smoking in Poland. This phenomenon may result from the anti-tobacco activities implemented under Polish Anti-tobacco Law [26,27]. The national tobacco control act is constantly amended to meet new tobacco control challenges such as e-cigarettes and heated tobacco products [27]. Nevertheless, further anti-tobacco activities are needed, especially targeted to high-risk groups, which will allow a steady decrease in the frequency of smoking in Poland [28,29]. 

In this study, men smoked more often than women, which is consistent with a previously reported survey [7,25]. Moreover, the highest prevalence of tobacco use was observed among participants with primary or vocational education, which is also in line with previously reported surveys [16,25]. We can hypothesize that lower-educated people are less aware of the health effects of tobacco use, which leads to a higher prevalence of smoking in this group. Moreover, the age of smoking initiation differed significantly depending on gender. Males were younger when they first tried cigarettes. An analysis of trends in smoking initiation in Europe over 40 years showed that smoking initiation during late adolescence declined for both sexes and in all European regions [30]. Preventing smoking initiation among adolescents is one of the key tobacco control activities. 

The most frequently used tobacco product was regular cigarettes. However, we observed that women smoked menthol or slim cigarettes significantly more often than men. It is believed that smokers may prefer menthol cigarettes to mask the bitter taste of nicotine [28,31,32,33,34]. An analysis of smoking behaviors revealed that menthol smokers are reporting greater subjective reward, satisfaction, and positive sensations in the throat from smoking compared to non-menthol smokers [31]. Moreover, there is a hypothesis on genetic vulnerability to menthol cigarette preference in women as a result of the genetic propensity to experience a heightened bitter taste [32]. According to smokers, menthol or slim cigarettes are also perceived as potentially less harmful than regular cigarettes [33,34]. The taste and smell of slim or menthol cigarettes, as well as their packaging, can be potential factors that lead women to reach for this form of tobacco products. We can hypothesize that the higher smoking prevalence of menthol or slim cigarettes among women than men may result from the fact that both menthol and slim cigarettes are seen as cooler and less risky. 

Hand-rolled cigarettes were the second most popular type of tobacco product smoked by the participants. In Poland, rolling tobacco is subject to a lower tax than conventional cigarettes, which makes hand-rolled cigarettes cheaper than conventional cigarettes. Such a high percentage of hand-rolled cigarettes (especially high among women) is probably due to economic factors and the price of rolling tobacco.

In this study, other forms of tobacco products such as pipe or shisha have not been used regularly by smokers, which results from the fact that in Poland, these products are not popular.

Novel tobacco products such as heated tobacco are a new form of nicotine delivery [12]. They are widely available in Poland since 2017 and advertised mainly on the Internet [12]. According to data from The Central Statistical Office, in 2018, 84.2% of households in Poland had access to the Internet. This is the first epidemiological study to assess the prevalence of heated tobacco use in the Polish population. Among the participants, 0.4% were current heated tobacco users. Data on the frequency of use of heated tobacco products are very limited [35,36,37]. The proportion of current heated tobacco users in Japan was consistently increasing, from 0.3% in 2015 to 3.6% in 2017 [35]. In Italy, ever use of HTPs was reported by 1.4% of Italians aged 15+ years [36]. In 2017, in the UK 1.7% of adults had ever used heated tobacco and 0.8% were current heated tobacco users [37]. Our study indicates that heated tobacco is not popular in Poland. One of the potential explanations of this observation may be the fact that the price of heated tobacco is much higher compared to e-cigarettes. Nevertheless, due to the intensive promotion of heated tobacco products, the frequency of their use requires constant monitoring.

According to the Eurobarometer 2017 survey, 2% of the EU population use e-cigarettes [7]. In this study, 1.4% of Poles were e-cigarette users. A similar percentage of e-cigarette users was observed in the study conducted in 2017 by Chief Sanitary Inspectorate with the same research methodology [16]. While the frequency of e-cigarette use in the general population is relatively low, e-cigarettes are gaining popularity among teenagers and young adults [13,38,39,40]. Among students aged 15–19 in Poland, the prevalence of current e-cigarette use increased from 2% in 2010–2011 to 11% in 2015–2016 [38]. Moreover, the percentage of dual users increased from 4% to 24% in this same observation period [38]. Among university students in Poland, the prevalence of current e-cigarette users varries between 2.9% and 3.5% [39,40]. A relatively low percentage of ever and current e-cigarette users in our study may result from the fact that e-cigarettes are the most popular in urban areas, especially in large cities. This study is based on a representative sample of Polish citizens, where inhabitants of rural areas or small/medium-sized cities predominate. 

Our study has some clinical implications. Women smokers exceed men in 30–39 year-olds, which could portend rising lung cancer rates in the future. Education on tobacco-related diseases including cancers in this age group should be particularly intensive. Moreover, there is a need for tobacco control activities dedicated to medium-aged (30–49 years), lower educated populations from medium-sized cities, where the prevalence of smoking was the highest. 

This study has several limitations. First, smoking status was defined based on self-reported data on tobacco use, so we cannot exclude the possibility of recall bias. The smoking status was not verified with biomarkers of tobacco smoking or environmental tobacco smoke exposures [41,42]. Nevertheless, in the case of interviewer-administered questionnaires, self-reported smoking status is described as an accurate measure tool [20,43]. Secondly, these studies assessed the prevalence of e-cigarette use in a nationwide representative sample of Poles aged 15+ years. It is known, that the group particularly vulnerable to use e-cigarette are adolescents and young adults. The frequency of using e-cigarettes in younger age groups may be higher than reported in the general population due to the significant share of old people in the demographic structure of Poland. Moreover, we cannot exclude reporting bias in the 15–19 year-old people. The legal smoking age in Poland is 18-years-old. So we can hypothesize, that people under the age of 18 were less likely to admit they were smoking. Nevertheless, this is the first epidemiological study conducted in 2019 on the prevalence of tobacco and e-cigarette use on a representative sample of Poles. The prevalence of tobacco use, especially novel tobacco products use, requires constant monitoring. 

## 5. Conclusions

This is the most up-to-date epidemiological study on the prevalence of tobacco and e-cigarette use in Poland. Compared to data from 2017, this study showed a decrease in the prevalence of smoking both for men and women. The prevalence of e-cigarette use, as well as heated tobacco use in a general population in Poland, is relatively low. The age group 30–49 years, lower educational level and living in a medium-sized city were significantly associated with current smoking status, and those groups should be recipients of tobacco control programs. Further tobacco control activities are needed to achieve smoke-free Poland in 2030.

## Figures and Tables

**Table 1:** Characteristics of the study population (*n* = 1011).

Variable	Total Sample	Women			*p*	Men			*p*
		Total	Smokers	Non-Smokers		Total	Smokers	Non-Smokers	
**Overall**	*n* = 1011 *n* (%)	*n* = 527 *n* (%)	*n* = 101 (19.2%) *n* (%)	*n* = 426 (80.8%) *n* (%)		*n* = 484 *n* (%)	*n* = 125 (25.8%) *n* (%)	*n* = 359 (74.2%) *n* (%)	
**Age (years)**									
15–19	63 (6.2)	32 (6.1)	1 (3.1)	31 (96.9)	< 0.01	31 (6.4)	0 (0.0)	31 (100.0)	< 0.01
20–29	169 (16.7)	83 (15.8)	8 (9.6)	75 (90.4)		86 (17.8)	21 (24.4)	65 (75.6)	
30–39	195 (19.3)	95 (18.0)	26 (27.4)	69 (72.6)		100 (20.7)	28 (28.0)	72 (72.0)	
40–49	153 (15.1)	76 (14.4)	15 (19.7)	61 (80.3)		77 (15.9)	27 (35.1)	50 (64.9)	
50–59	167 (16.5)	87 (16.5)	22 (25.3)	65 (74.7)		80 (16.5)	25 (31.3)	55 (68.7)	
60+	264 (26.1)	154 (29.2)	29 (18.8)	125 (81.2)		110 (22.7)	25 (22.7)	85 (77.3)	
**Marital Status**									
single	267 (26.4)	120 (22.8)	9 (7.5)	111 (92.5)	0.001	147 (30.4)	35 (23.8)	112 (76.2)	0.8
married	561 (55.5)	287 (55.0)	64 (22.3)	223 (77.7)		274 (56.6)	72 (26.3)	202 (73.7)	
divorced	67 (6.6)	39 (8.7)	12 (30.8)	27 (69.2)		28 (5.8)	9 (32.1)	19 (67.9)	
widowed	116 (11.5)	81 (15.1)	16 (19.8)	65 (80.2)		35 (7.2)	9 (25.7)	26 (74.3)	
**Place of Residence**									
rural	394 (39.0)	198 (37.6)	27 (13.6)	171 (86.4)	< 0.01	196 (40.5)	48 (24.5)	148 (75.5)	0.4
city up to 20,000 residents	131 (13.0)	70 (13.3)	14 (20.0)	56 (80.0)		61 (12.6)	12 (19.7)	49 (80.3)	
city between 20,000–100,000 residents	197 (19.4)	109 (20.7)	28 (25.7)	81 (74.3)		88 (18.2)	29 (33.0)	59 (67.0)	
city between 100,000–500,000 residents	172 (17.0)	90 (17.1)	22 (24.4)	68 (75.6)		82 (16.9)	23 (28.0)	59 (72.0)	
city above 500,000 residents	117 (11.6)	60 (11.4)	10 (16.7)	50 (83.3)		57 (11.8)	13 (22.8)	44 (77.2)	
**Educational Level**									
primary education	204 (20.2)	116 (22.0)	26 (22.4)	90 (77.6)	0.02	87 (18.0)	20 (23.0)	67 (77.0)	0.03
vocational education	249 (24.7)	97 (18.4)	27 (27.8)	70 (72.2)		153 (31.6)	52 (34.0)	101 (66.0)	
secondary education	336 (33.2)	181 (34.3)	32 (17.7)	149 (82.3)		155 (32.0)	37 (23.9)	118 (76.1)	
higher education	222 (21.9)	133 (25.2)	16 (12.0)	117 (88.0)		89 (18.4)	16 (18.0)	73 (82.0)	
**Occupational Status**									
active	628 (62.1)	292 (55.4)	51 (17.5)	241 (82.5)	0.3	336 (68.5)	94 (28.0)	242 (72.0)	0.1
passive	383 (37.9)	235 (44.6)	50 (21.3)	185 (78.7)		148 (31.5)	31 (20.9)	117 (79.1)	
**Financial Situation**									
good	232 (23.0)	116 (22.0)	16 (13.8)	100 (86.2)	0.2	116 (24.0)	25 (21.6)	91 (78.4)	0.2
moderate	497 (49.1)	263 (49.9)	57 (21.7)	206 (78.3)		234 (48.3)	60 (25.6)	174 (74.4)	
bad	282 (27.9)	148 (28.1)	28 (18.9)	120 (81.1)		134 (27.7)	41 (30.6)	93 (69.4)	

**Table 2:** Smoking characteristic of the study sample *n* = 1011.

Smoking Status		Total (*n*)	%	Women		Men		*p*
				*n*	% (95%CI)	*n*	% (95%CI)	
Current daily smokers		213	21.0	95	18.0 (15.0–21.5)	118	24.4 (20.8–28.4)	< 0.0001
Current occasional smokers		13	1.3	6	1.1 (0.5–2.5)	7	1.5 (0.7–3.0)	
Former smokers		108	10.7	37	7.0 (5.1–9.5)	71	14.7 (11.8–18.1)	
Non-smokers		677	67.0	389	73.8 (69.9–77.4)	288	59.5 (55.1–63.8)	
**The Type of Tobacco Products Smoked the Most**								
Regular cigarettes		163	76.5	61	64.2 (54.2–73.1)	102	86.4 (79.1–91.5)	< 0.0001
Menthol cigarettes		11	5.2	8	8.4 (4.3–15.8)	3	2.5 (0.9–7.2)	0.04
Slim cigarettes		13	6.1	11	11.6 (6.6–19.6)	2	1.7 (0.5–6.0)	0.003
Hand-rolled cigarettes		46	21.6	24	25.3 (17.6–34.8)	22	18.6 (12.7–26.6)	0.2
Heated tobacco products		4	1.9	2	2.1 (0.6–7.4)	2	1.7 (0.5–6.0)	0.8
Cigars		2	0.9	0	0.0 (0.0–3.9)	2	1.7 (0.5–6.0)	0.2
Cigarillos		0	0.0	0	0.0 (0.0–3.9)	0	0.0 (0.0–3.2)	0.9
Pipe		0	0.0	0	0.0 (0.0–3.9)	0	0.0 (0.0–3.2)	0.9
Shisha		2	0.9	0	0.0 (0.0–3.9)	2	1.7 (0.5–6.0)	0.2
Smokeless tobacco use		6	0.6	3	0.6 (0.2–1.7)	3	0.6 (0.2–1.8)	0.9
Start smoking in the past 12 months		25	11.1	12	11.9 (6.9–19.6)	13	10.4 (6.2–17.0)	0.7
**Number of Cigarettes Smoked Daily**								
	*n*	mean (± SD) median (range)		*n*	mean (± SD) median (range)	*n*	mean (± SD) median (range)	
Regular cigarettes	163	15.0 ± 6.215 (1–40)		61	14.7 ± 5.515 (3–25)	102	15.2 ± 6.619 (1–40)	0.4
Menthol cigarettes	11	9.0 ± 4.210 (2–20)		8	8.5 ± 3.115 (2–10)	3	10.8 ± 7.410 (5–20)	0.5
Slim cigarettes	13	11.8 ± 5.210 (1–20)		11	11.4 ± 5.210 (1–20)	2	14.4 ± 7.915 (10–20)	0.6
Hand-rolled cigarettes	46	16.5 ± 6.820 (3–40)		24	14.8 ± 6.920 (3–30)	22	18.4 ± 6.420 (6–40)	0.2
Heated tobacco sticks	4	10.7 ± 4.710 (6–15)		2	7.4 ± 2.58 (6–10)	2	15.0 15.0	0.9

95%CI—95-percent confidence interval; SD—standard deviation.

**Table 3:** Odds ratios (OR) and 95% confidence intervals (CI) for daily smoking to selected socioeconomic factors in a representative sample of men and women aged 15+ in Poland.

Variable	Total (*n*)	Daily Smokers		Univariate Logistic Regression		Multivariate Logistic Regression ^a^	
		*n*	%	OR	95%CI	OR	95%CI
**Gender**							
male	484	118	24.4	1.46 *	1.1–1.97	1.36	0.99–1.86
female	527	95	18.0	1.00	Reference	1.00	Reference
**Age (years)**							
15–19	63	1	1.6	0.07 **	0.01–0.48	0.06 **	0.01–0.41
20–29	169	27	16.0	0.74	0.45–1.24	0.95	0.55–1.62
30–39	195	49	25.1	1.33	0.86–2.07	1.76 *	1.10–2.82
40–49	153	40	26.1	1.40	0.87–2.24	1.77 *	1.08–2.91
50–59	167	43	25.7	1.50	0.89–2.22	1.58	0.98–2.54
60+	264	53	20.1	1.00	Reference	1.00	Reference
**Marital Status**							
single	267	40	15.0	0.68	0.38–1.18	-	-
married	561	129	23.0	1.15	0.70–1.87	-	-
divorced	67	20	29.9	1.74	0.88–3.46	-	-
widowed	116	24	20.7	1.00	Reference	-	-
**Place of Residence**							
rural	394	68	17.3	1.00	Reference	1.00	Reference
city up to 20,000 residents	131	25	19.1	1.1	0.66–1.70	1.16	0.69–1.97
city between 20,000–100,000 residents	197	55	27.9	1.89 **	1.26–2.84	1.90 **	1.25–2.89
city between 100,000–500,000 residents	172	45	26.2	1.71 *	1.12–2.63	1.79 *	1.15–2.79
city above 500,000 residents	117	20	17.1	0.98	0.57–1.70	1.15	0.65–2.05
**Educational Level**							
primary education	204	45	22.1	1.79 *	0.89–3.13	3.03 ***	1.75–5.26
vocational education	249	74	29.7	2.73 ***	1.71–4.37	2.87 ***	1.74–4.73
secondary education	336	64	19.0	1.49	0.93–2.38	1.77 *	1.09–2.87
higher education	222	30	13.5	1.00	Reference	1.00	Reference
**Occupational Status**							
active	628	134	21.3	1.05	0.77–1.44	-	-
passive	383	79	20.6	1.00	Reference	-	-
**Financial Situation**							
good	232	38	16.4	0.65	0.42–1.02	-	-
moderate	497	110	22.1	0.94	0.67–1.34	-	-
bad	282	65	23.0	1.00	Reference	-	-

^a^ Fully adjusted model including all statistically significant characteristics. *** *p* < 0.001; ** *p* < 0.01; * *p* < 0.05.
